# Understanding Genetic Diversity and Population Structure of a *Poa pratensis* Worldwide Collection through Morphological, Nuclear and Chloroplast Diversity Analysis

**DOI:** 10.1371/journal.pone.0124709

**Published:** 2015-04-20

**Authors:** Lorenzo Raggi, Elena Bitocchi, Luigi Russi, Gianpiero Marconi, Timothy F. Sharbel, Fabio Veronesi, Emidio Albertini

**Affiliations:** 1 Dipartimento di Scienze Agrarie, Alimentari e Ambientali, Università degli Studi di Perugia, Perugia, Italy; 2 Dipartimento di Scienze Agrarie, Alimentari ed Ambientali, Università Politecnica delle Marche, Ancona, Italy; 3 Department of Cytogenetics and Genome Analysis, Apomixis Research Group, Leibniz Institute of Plant Genetics and Crop Plant Research (IPK), Gatersleben, Germany; National Institute of Plant Genome Research, INDIA

## Abstract

*Poa pratensis* L. is a forage and turf grass species well adapted to a wide range of mesic to moist habitats. Due to its genome complexity little is known regarding evolution, genome composition and intraspecific phylogenetic relationships of this species. In the present study we investigated the morphological and genetic diversity of 33 *P*. *pratensis* accessions from 23 different countries using both nuclear and chloroplast molecular markers as well as flow cytometry of somatic tissues. This with the aim of shedding light on the genetic diversity and phylogenetic relationships of the collection that includes both cultivated and wild materials. Morphological characterization showed that the most relevant traits able to distinguish cultivated from wild forms were spring growth habit and leaf colour. The genome size analysis revealed high variability both within and between accessions in both wild and cultivated materials. The sequence analysis of the *trn*L-F chloroplast region revealed a low polymorphism level that could be the result of the complex mode of reproduction of this species. In addition, a strong reduction of chloroplast SSR variability was detected in cultivated materials, where only two alleles were conserved out of the four present in wild accessions. Contrarily, at nuclear level, high variability exist in the collection where the analysis of 11 SSR loci allowed the detection of a total of 91 different alleles. A Bayesian analysis performed on nuclear SSR data revealed that studied materials belong to two main clusters. While wild materials are equally represented in both clusters, the domesticated forms are mostly belonging to cluster P2 which is characterized by lower genetic diversity compared to the cluster P1. In the Neighbour Joining tree no clear distinction was found between accessions with the exception of those from China and Mongolia that were clearly separated from all the others.

## Introduction

*Poa pratensis* L., also known as Kentucky bluegrass, is a hardy, persistent, and attractive forage and turf grass species that is well adapted to a wide range of mesic to moist habitats. It belongs to the *Poaceae* (subfamily *Pooideae*, tribe *Poacea*), a family with more than 500 described species [1] and can hybridize with *P*. *secunda*, *P*. *arctica*, *P*. *alpina*, *P*. *nervosa*, *P*. *reflexa* and *P*. *palustris* [2] to form allopolyploids, many of which are facultative apomicts [3]. Hybridization is hypothetically facilitated by apomixis, as asexual reproduction provides an escape from the typical reproductive problems associated with unbalanced or mixed genomes. Bashaw and Funk [4] suggested that the great adaptiveness of this species is likely associated with its variable ploidy, ranging from 2n = 22 [5] to 2n = 147 [6], and its versatile mode of reproduction [7]. In fact the perpetuation of a single genotype through apomixis is an advantage in determining the *P*. *pratensis* circumpolar distribution [8] while sexual reproduction represent an important source of new genetic variation [3,9]. Studying a core collection of this species, Wieners and collaborators reported that the majority of the populations contained facultative apomicts (with a combination of reduced, zygotic and unreduced, parthenogenic embryo production), in addition to obligate sexual or obligate apomictic accessions [10].

An allopolyploid origin of *P*. *pratensis* is supported by its great polyploid and aneuploid variation, a consequence of which is that little is known regarding its evolution, genome composition and intraspecific phylogenetic relationships. Based on morphological, cytological and species diversity, it is generally accepted that *P*. *pratensis* originated mainly from Europe and Asia [11,12], followed by introduction to North America by European settlers in the Seventeenth Century [13]. According to other authors, some populations found in remote mountain meadows of western USA or the Appalachian mountains might be considered native [14,15] together with some ecotypes identified in the Rocky Mountains [16]. As karyology is by definition difficult in *Poa*, flow cytometry has been employed to investigate its genome size [10,17,18] and of other turfgrass species [19]. Since variation in DNA content may not be accounted for using different molecular markers, but may nonetheless have a significant role in determining the ultimate phenotype, this rapid and inexpensive method is useful in polyploids [10]. In addition genome size *per se* has adaptive significance influencing the phenotype by the expression of its genic content and by the physical effects of its mass and/or volume [20], and may additionally affect cross compatibility between genotypes. Finally, in angiosperms, DNA amount can correlate with a several relevant characters such as minimum generation time and ecological behaviour [18], and therefore deserves some attention in phylogenetic studies.

Kentucky bluegrass cultivars and accessions have previously been characterized using random amplified polymorphic DNA (RAPD) [21–23], inter-simple sequence repeat (ISSR) [24] and, only very recently, using simple sequence repeats markers (SSR) [25,26]. Although microsatellites are codominant markers, the difficulty in identifying the number of allele copies in polyploids limits their use. Here we attempt to overcome this hurdle by investigating the morphological and genetic diversity of 33 *P*. *pratensis* accessions using flow cytometry, sequencing of the *trn*L-F chloroplast region, in addition to nuclear and chloroplast microsatellite markers.

## Materials and Methods

### Plant materials, morphological characterization and total DNA extraction

**Fig 1:**
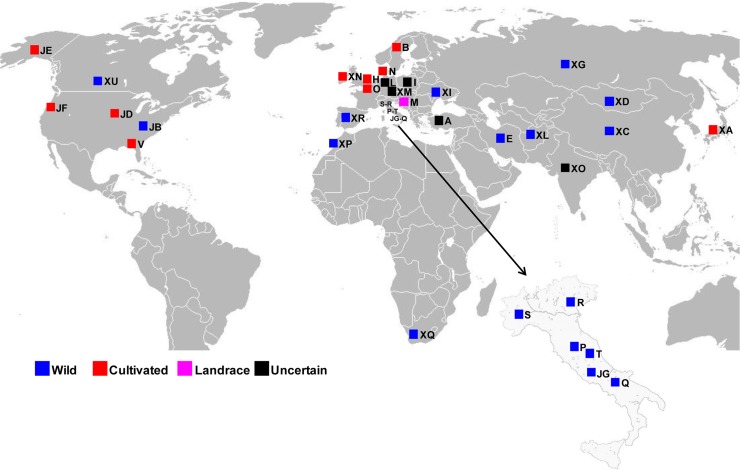
Geographical origin of *Poa pratensis* accessions. Geographical origin of the 33 accessions of *P*. *pratensis* used in this study, along with information of biological status. The figure was modified from Wikipedia and was for representative purposes only.

The phenotypic and genomic variability of 33 cultivated and wild accessions of *P*. *pratensis* from 23 different countries and 4 continents (Europe, Asia, America and Africa) were investigated (Fig 1; Table 1).

**Table 1:** List of studied accessions.

#	Accession name	Geographic origin	Sources	Type of accession	Mean DNA content
1	A	Turkey	PI 119684†	Uncertain improvement status	7.3 (14.2)
2	B	Sweden	PI 198075†	Cultivated	9.0 (4.9)
3	E	Iran	PI 227381†	Wild material	7.6 (12.3)
4	H	Netherland	PI 266209†	Prato	8.8 (4.7)
5	I	Poland, Lublin	PI 274645†	Uncertain improvement status	5.7 (8.9)
6	L	Germany	PI 283959†	Uncertain improvement status	9.2 (13.9)
7	M	Hungary	PI 298096†	Landrace	4.91
8	N	Denmark	PI 303057†	Cultivar	6.3 (9.7)
9	O	Belgium	PI 303061†	Cultivar	9.8 (5.5)
10	P	Italy, Tuoro (PG)	DSA3 3479*	Wild material	7.1 (10.3)
11	Q	Italy, Rionero Sannitico (IS)	DSA3 3481*	Wild material	6.4 (9.4)
12	R	Italy, Chioggia (VE)	DSA3 3484*	Wild material	6.8
13	S	Italy, Dormelleto (NO)	DSA3 3478*	Wild material	6.1
14	T	Italy, Serravalle del Chienti (MC)	DSA3 4678*	Wild material	8.0 (11.6)
15	V	United States, North Carolina	DSA3 5943*	PRINCETON	-
16	JB	United States, New York	W6 19117†	Wild material	6.9 (8.7)
17	JD	United States, Illinois	PI 578835†	Cultivar	6.8
18	JE	United States, Alaska	PI 562649†	NUGGET	9.7
19	JF	United States, Oregon	PI 601107†	MIDNIGHT	7.5 (12.0)
20	JG	Italy, Colle di Tora (RI)	DSA3 4676*	Wild material	-
21	XA	Japan	PI 438529†	Cultivar	7.5
22	XC	China	PI 618751†	Wild material	8.6 (13.2)
23	XD	Mongolia	PI 618768†	Wild material	6.87
24	XG	Russian Federation	PI 539048†	Wild material	6.74
25	XI	Ukraine	W6 21787†	Wild material	7.0 (11.0)
26	XL	Afghanistan	PI 221949†	Wild material	11.3
27	XM	Czech Republic	PI 286210†	Uncertain improvement status	6.4
28	XN	United Kingdom	PI 349801†	MONARCH	8.7
29	XO	India	PI 355956†	Uncertain improvement status	10.9 (12.7)
30	XP	Morocco	PI 436002†	Wild material	7.9
31	XQ	South Africa	PI 208179†	Wild material	8.5 (13.8)
32	XR	Spain	PI 423139†	Wild material	7.2 (11.8)
33	XU	Canada, Alberta	PI 387935†	Wild material	6.3 (11.3)

Accession name, geographic origin, GeneBank accession number, type of accession and mean somatic DNA content (pg) of the majority of plants sampled (in brackets the mean content of fewer plant within the same accession with a contrasting value).

*DSA3, Dipartimento di Scienze Agrarie, Alimentari e Ambientali.

† USDA GeneBank.

Seeds were sown in jiffy pots and, and 30 plants were grown for each accession. Total genomic DNA was extracted from 0.5 to 1 g of fresh leaf tissue per plant using the Genelute Plant genomic DNA miniprep kit (Sigma Aldrich) according to the manufacturer’s handbook. DNA concentration was assessed through optical density reading (DU650 spectrophotometer, Beckman) and confirmed by agarose gel electrophoresis.

For morphological characterization, 8 randomly chosen plants per accession were transplanted to the field in a randomized design and evaluated for: growth habit at flowering (scoring plants from 1 = erect to 9 = prostrate), rust resistance/tolerance (1 = r esistant/tolerant—9 = highly susceptible), leaf colour (1 = light green—9 dark green), stem length (mm), width and length of the flag leaf (mm), leaf texture (mm), spring plant regrowth (1 = slow—9 = very fast regrowth) and spring growth habit (1 = erect—9 = prostrate) after cutting. Some of the traits were evaluated according to the UPOV Guidelines [27]. Data were statistically analyzed using univariate one way ANOVA and multivariate discriminant analyses. A stepwise discriminant procedure was carried out in order to look at the most useful traits to use in a phenotypic characterization and for distinguishing populations on the basis of discriminant functions.

### Flow cytometry analysis of somatic tissue

Karyotyping was difficult and inaccurate due to the high numbers of chromosomes and variable ploidy levels, hence we estimated genome size through flow cytometric measurements of relative total DNA content in somatic tissues. Fifteen plants from each accession were grown in a greenhouse at the Leibniz Institute of Plant Genetics and Crop Plant Research (IPK, Gatersleben, Germany), and an average of seven plants were randomly selected for flow cytometry. Nuclei were collected from young leaves of single plants by manual chopping them with a razor blade in the following staining buffer: 100 mM Tris-HCl, 5.3 mM MgCl_2_, 86 mM NaCl, 0.03 mM sodium citrate, 7.3 mM Triton X-100, 3 μM 4‘-6-diamidino-2-phenylindole (DAPI), pH 7.0. After filtration through nylon tissue (30-μm mesh) to remove large debris the tubes were stored in the dark on ice for 1 to 4 h before measurement [28]. Fluorescence intensity of DAPI–stained nuclei was measured on a Ploidy Analyser PA (Partec, Münster, Germany).

For each sample the standardized 2C DNA content was obtained by using the following equation: position of sample main peak times position of the internal control main peak (a triploid *Boechera holboellii* genotype) divided by mean position of the peak of the internal control. The repeated measure of the internal control over multiple days allowed to standardize the main peak position of each *P*. *pratensis* samples. The final DNA quantification in pictograms was then estimated through a simple linear regression based on four accessions (PI 286210, PI 298096, PI 355956 and PI 601107) characterized by Wieners and collaborators [10] and common in both studies (R^2^ = 0.91, P<0.05). Since individual plants within the same accession might show different somatic tissue DNA content, particularly when multiple reproductive pathways occur, standard errors would be misleading and were therefore not calculated [10]. Differences between wild, cultivated and accessions with uncertain improvement status were analysed by one way ANOVA.

### *trn*L-F region amplification and sequencing

A fragment of the chloroplast *trnL-F region* was PCR amplified from DNA extracted from 70 randomly chosen individuals representing all 33 collected accessions using primers “c” (CGAAATCGGTAGACGCTACG) and “f” (ATTTGAACTGGTGACACGAG) of Taberlet and collaborators [29]. Ten μl of the PCR products were electrophoresed on a 1.5% (w/v) agarose gel and visualized by ethidium bromide staining. Successful amplifications were purified using Qiaquik PCR purification Kit (Qiagen) following manufacture instructions. Sequencing reactions were performed for both DNA strands using the ABI PRISM BigDye Terminator Cycle Sequencing Ready Reaction Kit (Applied Biosystems) on an ABI 3700 genetic analyser according to the manufacturer’s instruction. Amplification primers used for PCR were also used for sequencing. All sequence alignments were carried out using MEGA version 4 software [30].

### *trn*L-F region CAPS marker development and analysis

Since only one Single Nucleotide Polymorphism (SNP) was detected in the chloroplast sequenced region (see below) we developed a Cleaved Amplified Polymorphic Sequences (CAPS) marker to test the allelic condition at the locus. PCR primers were developed using PRIMER 3 [31] from multiple alignments of sequences obtained in this study and those retrieved from public databases, and the restriction endonuclease was selected according to the CAPS differential cleavage sites. The allelic condition for the developed CAPS marker was tested on eight individuals randomly chosen from each accession (hereafter population) for a total of 264 individuals. PCR amplifications were carried out using the developed cpCAPfor and cpCAPrew (Table 2) in a 20 μl volume containing 25 ng genomic DNA, 1× PCR buffer (Invitrogen), 200 μM dNTPs, 0.5 μg of Bovine Serum Albumin (BSA), 10 pMol of the cpCAPsf and cpCAPsr primers, and 1 U of *Taq* DNA polymerase (Invitrogen).

**Table 2:** Characteristics of CAPS primer developed.

Primer name	Primer sequence (5'-3')	Ta (°C)	Frag. lenght (bp)
cpCAPfor	GCAATCCTGAGCCAAATCCGTGTT	52	429
cpCAPrew	ATGGGACTCTCTCTTTGTCCTCGT	54	

Primer name, sequence, annealing temperature (Ta) and Fragment (Frag.) length in base pair (bp) of the CAPS primer developed.

All reactions were performed using the following cycling parameters: 94°C for 5 min, followed by 38 cycles of 94°C for 30 s, 51°C for 30 s, and 72°C for 45 s. Aliquots of 11 μl PCR product were digested with 2 units of *Bsm*I in 1× Buffer R for 2 h at 37°C in a total volume of 25 μl. After digestion, 15 μl of the reaction was electrophoresed on a 2% (w/v) agarose gel and visualized by ethidium bromide staining.

### Nuclear and chloroplast SSR analyses

A total of 11 nuclear and two chloroplast single sequence repeat (SSR) markers were used to genotype the 264 *P*. *pratensis* samples tested for the CAPS marker allelic condition (Table 3).

**Table 3:** List of nuclear and chloroplast SSR primers.

Primer name	Species/family	DNA type	Primer sequence	Total amplified alleles	
Poa287 For	*Poa alpina*	nuclear	5'-AAGCAGCTCGGTCTCTTTTG-3'	17	*
Poa287 Rev	*Poa alpina*	nuclear	5'-TGCAGGCACCCAGATAAAGT-3'		*
Poa290 For	*Poa alpina*	nuclear	5'-GGAAACCCAGAATCAGCAAA-3'	18	*
Poa290 Rev	*Poa alpina*	nuclear	5'-CCATATTGGGAGTTCCTCATC-3'		*
Poa310 For	*Poa arachnifera*	nuclear	5'-AGTCCCTTTACGGTTTACCT-3'	-	*
Poa310 Rev	*Poa arachnifera*	nuclear	5'-CCATATACCGGTGTCTTCTC-3'		*
Poa406 For	*Poa arachnifera*	nuclear	5'-CTACCTGTCAGTCTCAAGGC-3'	-	*
Poa406 Rev	*Poa arachnifera*	nuclear	5'-ATACATTCCCGTAAATGGA-3'		*
Poa414 For	*Poa arachnifera*	nuclear	5'-GTGGATCAGAATGGGTGCTT-3'	10	*
Poa414 Rev	*Poa arachnifera*	nuclear	5'-AAGCAGGCATGGTCAGAATC-3'		*
Poa416 For	*Poa arachnifera*	nuclear	5'-AGATGCAATAAGGCGAATGG-3'	3	*
Poa416 Rev	*Poa arachnifera*	nuclear	5'-AGGGTCTTGGGCTTGTTCTT-3'		*
Poa420 For	*Poa arachnifera*	nuclear	5'-GGAAGTCCGCACTTGACAAT-3'	2	*
Poa420 Rev	*Poa arachnifera*	nuclear	5'-GCGCTTGAAGCAAACAATCT-3'		*
Poa410 For	*Poa arachnifera*	nuclear	5'-TACAGAGGCCAGGGTTTCAC-3'	10	*
Poa410 Rev	*Poa arachnifera*	nuclear	5'-AGCTTGGAGCATGTCAGAGG-3'		*
Poa415 For	*Poa arachnifera*	nuclear	5'-GTCACCTTGAAGCACGGTTT-3'	18	*
Poa415 Rev	*Poa arachnifera*	nuclear	5'-TCTAGCTGCAGGTGAGATGC-3'		*
AE5 For	*Poa pratensis*	nuclear	5'-TCTCCCTCATAACCTAACAGAATTA-3'	12	†
AE5 Rev	*Poa pratensis*	nuclear	5'-GAGAAGGCATCTGTAAATGATACAG-3'		†
AE11 For	*Poa pratensis*	nuclear	5'-TCTAGGAAGAAGGTGATATAA-3'	1	†
AE11 Rev	*Poa pratensis*	nuclear	5'-GCCTCGGGCAGCCCCACG-3'		†
*rpo*C2 For	*Poaceae*	chloroplast	5'-TTATTTATTTCAAAGCTATTTCGG-3'	1	+
*rps*2 Rev	*Poaceae*	chloroplast	5'-AATATCTTCTTGTCATTTTTTCC-3'		+
*atp*B For	*Poaceae*	chloroplast	5'-GATTGGTTCTCATAATTATCAC-3'	4	+
*rbc*L Rev	*Poaceae*	chloroplast	5'-TATTGAATTAACTAATTCATTTCC-3'		+

Primer name, species or family for which primer were originally developed, DNA type amplified, primer sequences and total amplified alleles of nuclear and chloroplast SSR primer used in the study.

* Bryan K Kindiger, Agricultural Research Service (USDA), personal communication.

† Albertini et al. 2003.

+ Provan et al. 2004.

Concerning microsatellites derived from *P*. *alpina* and *P*. *arachnifera*, PCR amplification was performed in a total volume of 20 μl containing 30 ng genomic DNA: 1× PCR buffer (New England Biolabs), 200 μM dNTPs, 3,5 pM of the Forward and Reverse primers and 0,75 U of *Taq* DNA polymerase (New England Biolabs). Since primer were not originally developed for *P*. *pratensis* a touchdown PCR method was used to optimize yield of amplified DNA and amplification specificity [32]. The reactions were performed using the same cycling parameters: 94°C for 3 min, followed by 10 cycles of 92°C for 30 s, 58°C for 40 s (decreasing of 0.7°C for each cycle) and 72°C for 60 s and 25 cycles of 92°C for 30 s, 50°C for 30 s and 72°C for 60 s. All others amplifications were performed in a total volume of 12 μl containing 15 ng genomic DNA: 1× PCR buffer (Sigma Aldrich), 1.5 mM MgCl_2_, 200 μM dNTPs, 4 pM of the Forward and Reverse primer and 0,75 U of *Taq* DNA polymerase (Sigma Aldrich). The following PCR cycling parameters were used: 94°C for 3 min, 35 cycles of 94°C for 30 s, 56°C for 30 s, 72°C for 30 s, and an extension final step of 72°C for 20 min. Amplification products were resolved and analyzed with an ABI 3100 Genetic Analyzer (Applied Biosystems), using Dye Set G5 (ABI) labelled primers. Size calculation and data record of generated amplicons were performed using GeneMapper 3.0 software (Applied Biosystems).

### Population structure analysis

Considering variable ploidy in *P*. *pratensis* and the fact that it was not possible to infer relative dosage of PCR products, nuclear SSR alleles were scored as dominant markers (i.e. presence/absence of amplicons). We carried out the analysis by using the software STRUCTURE ver. 2.3.4 [33,34] to investigate the genetic population structure of the 264 *P*. *pratensis* individuals from 33 populations. This software has been successfully used to evaluate genetic structure of individuals on different apomictic species, such as for example those belonging to *Crataegus*, *Brachiaria*, *Panicum*, *Taraxacum*, and *Paspalum* genera [35–39]. The approach uses a Markov Chain Monte Carlo (MCMC) algorithm to group individuals into populations (clusters) on the basis of multilocus genotype data [33,34]. In this study the procedure that accounts appropriately for the genotypic ambiguity inherent in dominant markers [40] was followed. The number of clusters (K) was estimated by carrying out ten independent runs for each K (from 1 to 10), using 30,000 MCMC repetitions and 30,000 burn-in periods, assuming correlated allele frequencies (*Run 1*). No *a priori* population information was used. The percentage of membership (q) of each individual in each of the inferred K clusters was computed by one additional STRUCTURE run for 100,000 MCMC repetitions and 100,000 burn-in periods (*Run 2*).

Two different approaches were used to set the number of clusters (K), the *ad hoc* statistic ΔK [41] and the change in H’ (average symmetric similarity coefficient) from CLUMPP software [42]. The latter indicates the goodness of fit to assign each individual to a given cluster(s), consistently across STRUCTURE independent runs. For each K, the results of *Run 2* were compared with those obtained by merging the 10 initial STRUCTURE simulations (*Run 1*) using the CLUMPP software. CLUMPP was used with the Greedy Algorithm method.

Data on chloroplast DNA variation for both cpSSR (*atpB-rbcL*) and CAPS marker (*trn*L-F) were used to classify individuals into different groups on the basis of their relative alleles.

### Genetic diversity and phylogeographic analysis

Genetic diversity analysis was carried out using i) the nuclear SSR raw data of the 33 populations and ii) the clusters identified by the software STRUCTURE. For the former analysis, the gene diversity Hj, analogous to the unbiased expected heterozygosity He [43], was computed from allele frequencies estimated following the approach of Lynch & Milligan [44]. The estimates were obtained by AFLPSURV [45], a software using a Bayesian method based on a non-uniform prior distribution of the allele frequencies [46], and able to produce unbiased estimates in dominant markers [47]. For the latter analysis, the F_K_ parameter for each of the K clusters [34] was estimated using the software STRUCTURE. The parameter F_K_ represents the estimated drift from the inferred common ancestor of all populations, thus similar to F_ST_ but specific for each cluster and expected to be proportional to the divergence from a common ancestral population. A low F_K_ value indicates little drift away from the ancestral state. The Tukey-Kramer HSD (Honestly significant difference) procedure was used to test for differences in gene diversity estimates among the 33 populations, while the Wilcoxon signed-ranks non-parametric test for two groups, arranged for paired observations, (i.e. one pair of estimates for each locus) [48], and Bonferroni correction for multiple comparisons was used to test for significant differences among clusters identified by STRUCTURE analyses.

Finally, to further shed light on the relationships among populations, a consensus NJ tree based on F_ST_ estimates between the 33 populations was obtained; 500 matrices of F_ST_ were computed by bootstrapping over loci by using AFLP-SURV [45] on the basis of allele frequencies estimated by the Zhivotovsky [46] approach. The CONSENSE procedure implemented in the PHYLIP software package [49] was used to infer bootstrap values.

### Correlation tests

The association between the clusters, as obtained by a genetic diversity analysis at nuclear SSR, and the chloroplast data, for both cpSSR (*atpB-rbcL*) and CAPS marker (*trn*L-F), was tested by analysis of contingency tables with the likelihood ratio chi-squared (χ^2^) test. To evaluate whether the nuclear DNA content was significantly associated with membership coefficients of identified K clusters and alleles of chloroplast markers a bivariate and one way analysis were performed. Finally, a correlation between morphological traits and DNA content was carried out on mean population values. All the test were performed using the JMP 8.0 software (SAS Institute Inc. 2008, Cary, NC, USA).

## Results

### Morphological characterization

The phenotypic characterization of 32 *P*. *pratensis* populations (no plants of XD accession survived transplanting) showed highly significant differences for each of the examined traits (P<0.001; Table 4).

**Table 4:** Morphological characterization results.

	Growth habit at flowering	Infloresc. Pigment.	Rust tolerance	Leaf colour	Stem lenght	Flag leaf width	Flag leaf lenght	Leaf texture	Spring growth	Spring growth habit
M	5.86	6.14	4.86	3.71	67.14	4.53	54.00	5.01	4.67	6.00
A	4.86	2.43	3.29	6.43	66.00	3.49	47.86	3.47	3.86	3.86
I	6.00	4.50	2.88	7.25	46.33	4.07	46.14	3.64	3.25	6.25
L	5.57	4.00	4.57	6.00	44.00	3.19	27.29	3.86	4.71	5.00
XM	5.25	5.00	6.50	5.75	48.25	3.78	68.25	3.75	4.00	6.00
XO	6.00	1.00	6.50	5.00	53.00	4.50	49.00	4.00	2.50	5.00
B	5.00	2.00	3.83	5.50	55.80	4.10	50.80	4.23	3.00	5.67
H	7.33	3.33	7.00	4.33	30.67	3.20	40.33	4.37	3.67	7.00
JD	5.75	2.50	5.00	5.13	50.83	3.70	42.14	4.29	4.38	6.25
JE	7.17	5.00	4.33	5.50	15.00	2.83	22.33	3.93	1.60	5.40
JF	6.80	-	4.20	6.80	10.00	4.40	36.33	3.32	2.00	8.20
N	3.38	3.75	6.13	5.00	73.50	3.58	59.25	3.58	3.63	3.75
O	6.75	3.63	5.88	5.13	37.00	2.91	33.75	3.59	3.25	6.50
V	7.29	3.86	2.86	8.57	29.14	3.20	34.00	3.89	3.29	6.71
XA	6.33	3.20	3.83	5.50	56.00	3.90	49.20	4.05	3.67	4.67
XN	5.50	4.40	4.67	5.00	48.20	3.86	34.80	5.17	2.17	6.20
E	5.80	3.00	6.80	4.20	57.00	2.76	26.00	3.52	4.00	1.40
JB	5.60	2.40	6.80	5.00	45.80	3.06	35.40	2.92	5.20	2.20
JG	5.71	4.67	4.00	6.00	56.00	2.77	44.86	4.24	6.29	5.29
P	4.38	3.88	5.75	5.75	61.63	2.39	32.50	2.90	5.75	2.50
Q	4.67	1.50	4.13	6.00	68.63	2.76	27.38	2.85	5.38	2.50
R	3.50	3.00	3.38	6.75	56.00	2.41	25.50	2.75	7.25	1.00
S	4.14	3.50	3.29	5.86	51.33	3.92	53.17	4.36	5.29	3.86
T	5.13	3.25	7.63	4.88	62.13	2.19	20.38	3.63	6.75	2.75
XC	7.00	2.00	7.00	4.00	42.33	5.93	53.00	5.57	1.67	5.67
XG	5.43	3.33	4.71	5.00	64.33	2.95	42.00	3.36	5.71	2.71
XI	5.00	6.50	5.29	5.29	50.17	2.25	37.50	3.33	4.43	4.43
XL	6.00	2.00	6.40	4.60	59.67	3.60	41.67	4.18	3.80	3.80
XP	7.00	2.33	6.33	5.00	51.33	4.10	39.67	3.80	2.33	3.00
XQ	4.13	2.13	6.50	4.88	41.75	2.61	40.63	3.59	3.25	4.50
XR	6.33	1.83	5.29	5.00	36.43	3.16	37.86	2.51	2.71	5.29
XU	5.43	2.14	6.00	5.29	52.43	4.24	47.00	3.57	3.71	5.00
*SE*	*0*.*660*	*0*.*691*	*0*.*968*	*0*.*451*	*8*.*910*	*0*.*568*	*9*.*354*	*0*.*466*	*0*.*903*	*0*.*800*
Cultivated	5.97	3.48	4.73	5.68	45.88	3.53	41.63	4.01	3.15	5.92
Wild	5.08	3.02	5.43	5.33	54.12	3.01	36.58	3.46	4.86	3.45
*SE*	*0*.*231*	*0*.*257*	*0*.*305*	*0*.*175*	*3*.*046*	*0*.*184*	*2*.*847*	*0*.*151*	*0*.*299*	*0*.*288*

Morphological characterization of 32 accessions of *P*. *pratensis* and means of grouped accessions according to the status as reported in Table 1.

When populations of known origin were separated into cultivated and wild groups, traits such as spring growth habit, leaf colour, leaf texture and spring regrowth, showed significant differences (P<0.05), with cultivated accessions more prostrate, more tolerant to rust, having a darker green colour and slower spring regrowth as compared to wild accessions (Table 4). Stem length, a trait normally used to characterize a variety for registration procedures, was higher in wild compared to cultivated populations. Leaf texture and width of the flag leaf were higher in cultivated accessions, while no statistical significances were found for inflorescence pigmentation and flag leaf length (Table 4).

**Fig 2:**
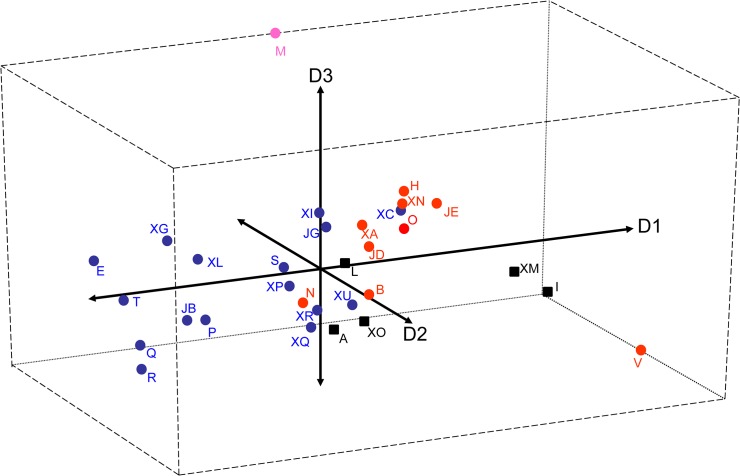
Results of stepwise discriminant analysis. Centroids of the 32 successfully characterized *P*. *pratensis* accessions plotted according to the first three discriminant functions (fun 1 = 1, fun 2 = 2 and fun 3 = 3).

The stepwise discriminant procedure included all recorded traits and increased in each step the averaged scored canonical correlation (P<0.001). Out of the ten possible discriminant functions found, the first eight were highly significant, with the first three able to explain over 66% of the total variability (29, 23 and 14%, respectively). The first discriminant function was highly and positively correlated with spring growth habit (0.61), the second negatively correlated with leaf colour (-0.70) and the third positively correlated with pigmentation of the inflorescence (0.64) and spring regrowth (0.60). The centroids of the class means, plotted according to the first three functions, showed that cultivated and wild accessions were clearly separated by the first discriminant function (cultivated accession being more prostrate than wild); no clear cut was observed according to the second discriminant function, although the leaf colour of some cultivars (B, N and V) were generally darker than many wild accessions (E, XC, XI). The third function was able to separate the cultivated from the uncertain origin basically for the presence of inflorescence pigmentation (Fig 2). Accession V, the cultivar Princeton from the USA, was unique for its prostrate growth habit and dark green colour of the leaves. Similar characteristics were shown by accession I and XM, both of uncertain improvement status and from East European countries (Poland and Czeck Republic, respectively). Also landrace M from Hungary clustered apart and was characterized by a light green colour, early spring regrowth and intense inflorescence pigmentation.

### Genome size analysis

**Fig 3:**
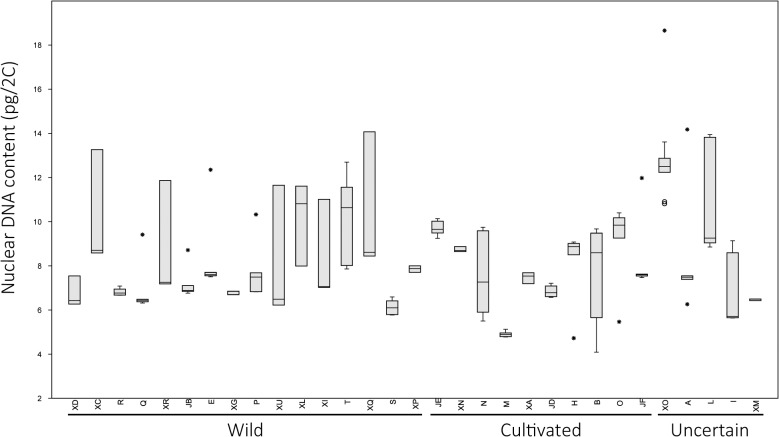
Nuclear DNA content. Box plot representation of Nuclear DNA content (pg/2C) of successfully characterized *P*. *pratensis* accessions. The “whiskers” are drawn from the top of the box up to the largest data point less than 1.5 times the box height from the box (the "upper inner fence"), and similarly below the box. Values outside the inner fences are shown as circles, values further than 3 times the box height from the box are shown as stars.

The total variation in terms of DNA content among 176 *P*. *pratensis* genotypes (JG and V were not included) ranged between ~4.08 pg (accession B) to ~18.66 pg (XO) (Fig 3), while the mean DNA content of each accession ranged from ~4.91 (accession M) to ~12.69 pg (XO) (Table 1). The overall data reveal high variability both within and between populations (Fig 3).

Uniform DNA content within accessions were found in M, R, S, JD, JE, XA, XD, XG, XL, XM and XP, while all others showed two distinct groups. DNA content of plants that showed contrasting values compared to the majority of plants of the same accession is reported in brackets in Table 1 last column.

Considering only the somatic DNA content of the majority of plants present in each accession, no significant differences were detected between cultivated, wild and populations of uncertain improvement status. The distribution of DNA content of the accessions clearly shows a bi-modal distribution, with two major peaks, around 6–7 and 9–10 pg values (S1 Fig). According to the relationship between DNA content and chromosome number, developed for Kentucky bluegrass by Eaton and collaborators [18], the most common chromosome numbers in the collection are between 52 and 60 followed by those between 60 and 68. DNA content was correlated with some morphological traits: in cultivated materials it was negatively correlated with the spring regrowth (-0.66) and with the length and width of the flag leaf (-0.67 and -0.71, respectively), while in wild populations it was negatively correlated with the intensity of the green colour (-0.56) and positively correlated with the rust tolerance (0.54) (all P<0.05).

### Molecular characterization

**Fig 4:**
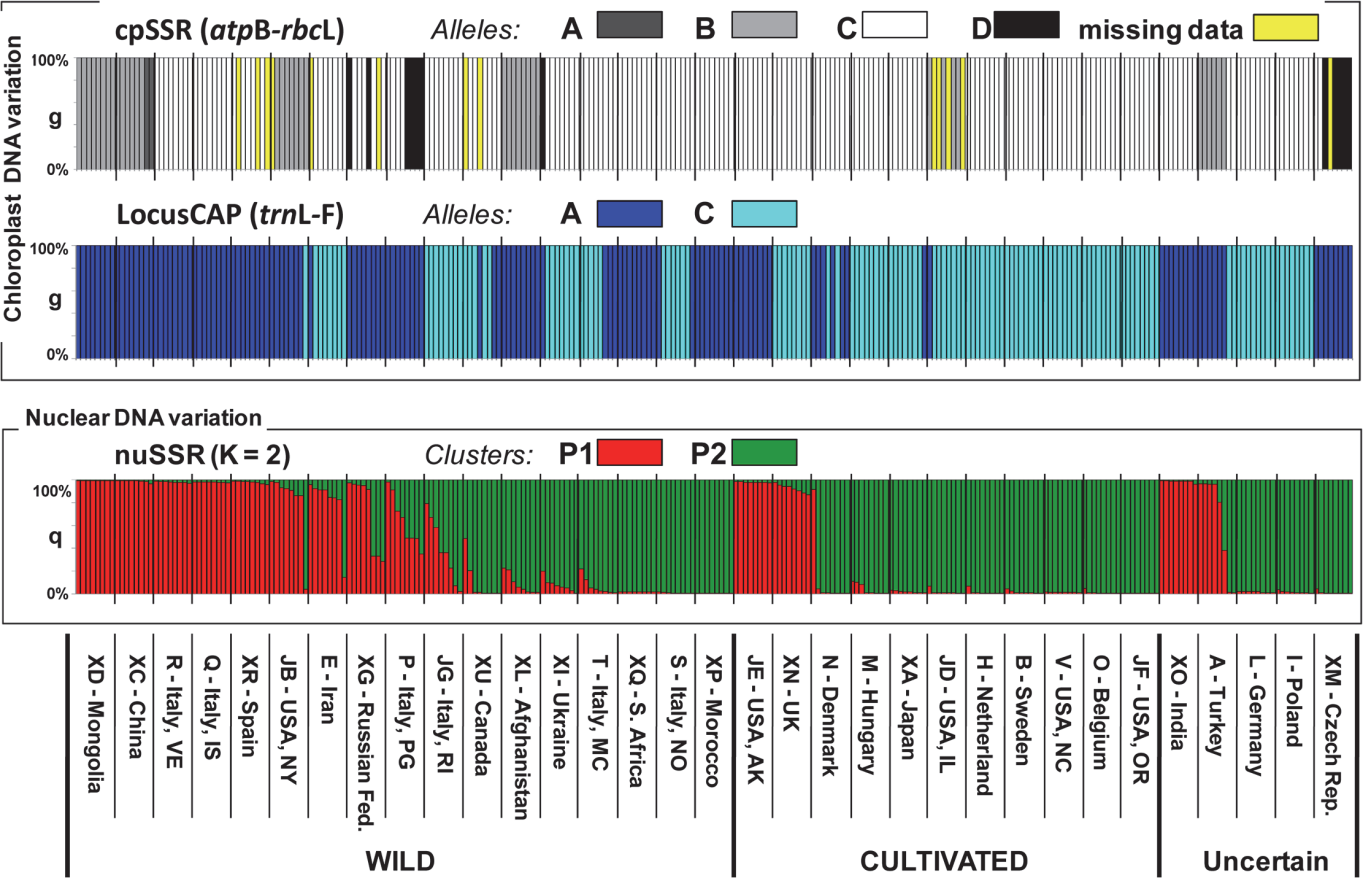
Molecular characterization results grouped by genotypes. Molecular characterization of the 264 genotypes used: chloroplast data, each individual is represented by a vertical line coloured according to the allele type of the relative locus; nuclear data, each individual is represented as a vertical line, partitioned into K coloured segments, which represent the individually estimated membership fractions in the two clusters identified.

Using two chloroplast SSR primer sets, 5 discernible DNA fragments were obtained: the *atp*B-*rbc*L locus was polymorphic with four different alleles (A, B, C and D, respectively) (Fig 4, chloroplast DNA variation part) while *rpo*C2-*rps*2 was monomorphic and therefore not included in successive analysis.

The portion of the sequenced *trn*L-F region yielded sequences ranging from 561 to 566 bp. The alignment of the produced sequences with those available in the NCBI database (AY061951 and AY504641) revealed the presence of only 2 polymorphisms: i) a 5 bp indel (AATAT) at position 328 bp found only in Mongolian (XD) and Indian (XO) populations, and ii) a transversion from adenine to cytosine at position 359 bp (S2 Fig). In the 264 genotypes tested with the CAPS marker developed in this study, adenine type incidence was 51.1% (135 genotypes) while cytosine type incidence was 48.9% (Fig 4, chloroplast DNA variation part). According to SNP allelic state, the 33 studied populations were grouped in: i) adenine containing (13 populations, 39.4%); ii) cytosine containing (10 populations, 30.3%) and iii) adenine and Cytosine containing group (10 populations, 30.3%).

Scorable amplicons were produced for 9 of the 11 nuclear SSR, as loci Poa310 and Poa406 produced unclear electrophoretic profiles (Table 3). Locus SSR AE11 was monomorphic, while the remaining loci were polymorphic with a maximum of 18 alleles (for Poa290 and Poa415) and an average of 10.1 (Table 3). The number of alleles per genotype varied from a minimum of 1 (as for several genotypes at Poa001, Poa002, Poa290, Poa410, Poa414 and AE11 loci) to 10 different alleles. A total of 91 different alleles were detected.

### Population structure, genetic diversity and phylogeographic analysis

The *ln likelihood* estimates increased progressively at increasing values of K (S3A Fig). On the basis of the method of Evanno and collaborators [41] (S3B Fig), the genotypes were split into K = 2 groups (hereafter referred to as cluster P1 and P2). A lower peak of ΔK was identified at K = 5 (S3B Fig). The highest H’ value from CLUMPP was that for K = 2 (H’ = 0.76), confirming the indication obtained from the Evanno’s approach. As shown in S3C Fig, the H’ estimates for other K values were comprised between 0.59 at K = 3 and 0.45 at K = 7, thus not supporting any lower hierarchical structure. The percentages of memberships (q values) at K = 2 were then recalculated by a new run based on 100,000 repetitions and burn-in periods. The q values so obtained were consistent with those of CLUMPP (H’ = 0.82), hence we used those from the STRUCTURE’s run. A threshold value of q = 0.70 was used to assign genotypes to one of the inferred K clusters (Fig 4).

**Fig 5:**
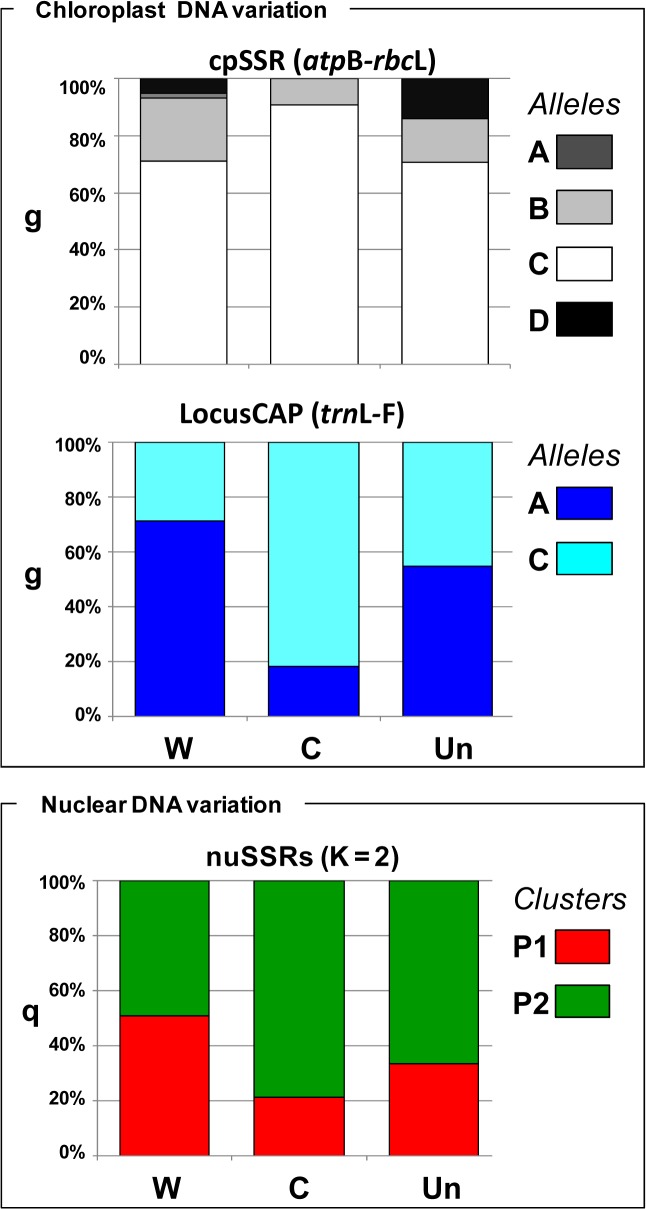
Molecular characterization results grouped by accession status. Molecular characterization of the *P*. *pratensis* genotypes grouped for biological status: wild (W), cultivated (C) and Unknown (Un). Chloroplast DNA variation: each group of individuals is represented as a vertical line coloured according to the percentage of different alleles detected at the relative locus. Nuclear DNA variation: each group of individuals is represented as a vertical line, partitioned into K coloured segments, according to the percentage of estimated membership in the two clusters identified.

At K = 2 the cluster P1 included 93 genotypes, 63 (67,7%), 17 (18.3%), and 13 (14%) were wild, cultivated and of uncertain improvement status, respectively. Seventy-eight (83.9%) and 15 (16.1%) P1 genotypes showed the ‘A’ allele and the ‘C’ allele of the CAP locus, respectively. Cluster P2 included more genotypes (158), and of these 61 were wild (38.6%), 71 cultivated (44%) and 26 had uncertain biological status (16.5%). Most of them (69%) carried the ‘C’ allele, with the remaining (31%) carried the ‘A’ allele of the CAP locus. Thirteen genotypes were admixed (0.30<q<0.70), among them 12 were wild and one with a doubtful biological status (Fig 4). Wild materials were assigned to both P1 and P2 clusters (mean q_P1_ = 0.51 and mean q_P2_ = 0.49), while cultivated genotypes were mostly assigned to cluster P2 (mean q_P2_ = 0.79; Fig 5).

At the chloroplast level for the locus CAP (*trn*L-F) we found contrasting results for wild and cultivated materials, with the wild mostly represented by genotypes carrying the ‘A’ allele (71%) and the cultivated mostly represented by genotypes carrying the ‘C’ allele (82%). Finally, the DNA content was not significantly correlated with q_P1_ values and the two chloroplast loci. Cluster P1 was characterized by a significantly higher genetic diversity as compared to cluster P2 (P<0.05, Wilcoxon signed-ranks non-parametric test and Bonferroni correction; Table 5).

**Table 5:** Genetic diversity comparison.

Cluster (K = 2)	N	Hj^†^	SD_Hj_	F_K_ ^‡^
P1	93	0.20A	0.017	0.09
P2	158	0.17B	0.017	0.18
MIX^1^	13	0.21A	0.015	na

Genetic diversity and FK estimates for the clusters identified by STRUCTURE analysis. The K = 2 partition was considered.

^1^Admixed genotypes (0.30 < q_P1_ < 0.70) were grouped as MIX population.

† Hj values followed by different letters are significantly different at P<0.05.

‡ na, not applicable.

Moreover, cluster P1 showed a lower F_K_ estimate (0.09) as compared to cluster P2 (F_K_ = 0.18). A significant F_ST_ value was found between P1 and P2 clusters (F_ST_ = 0.09, P< 0.05). All populations showed similar level of gene diversity (mean Hj = 0.22), with few exceptions (Hj of O population was significantly higher than that of Q, JF, XP and XQ populations; S1 Table).

**Fig 6:**
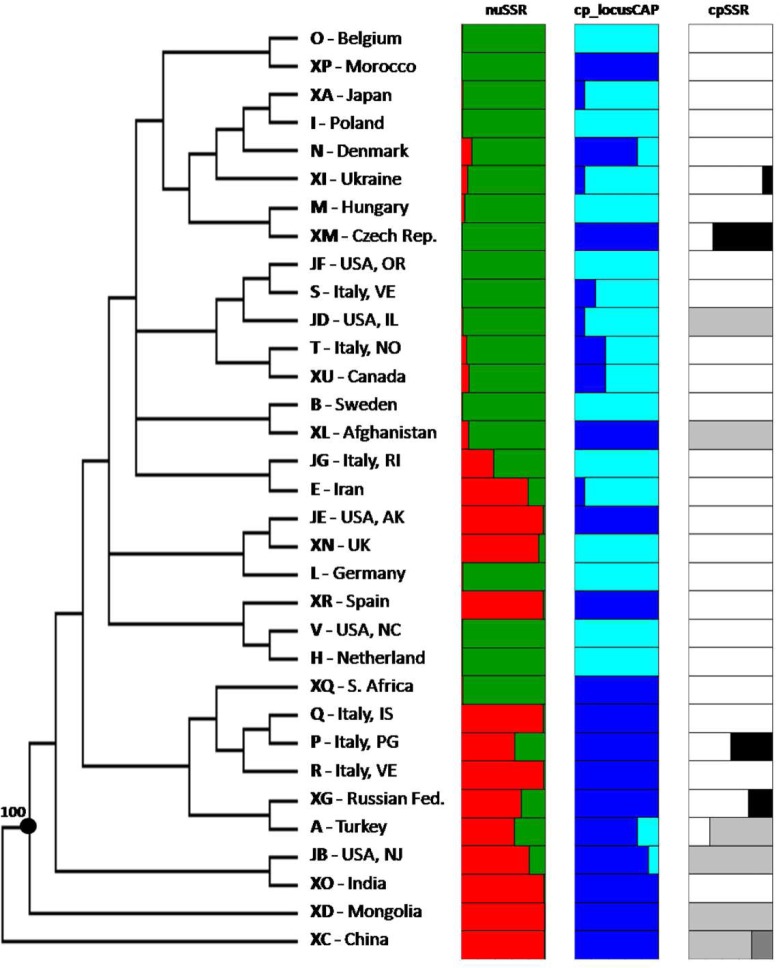
Cluster analysis results. Neighbor-joining tree based on nuclear SSR data compared with the percentage of membership for the 33 accessions computed for the two clusters identified and the allelic state of the two chloroplast loci. Only bootstrap values higher than 50% are showed.

The NJ tree (Fig 6) revealed the relationships between the 33 populations analysed. No clear distinction was found between them, indeed bootstrap values resulted lower than 50% for almost all the nodes of the tree. However, it is worth noting that accessions from China and Mongolia clustered apart from the other populations (100% of bootstrap value). China (XC) and Mongolia (XD) populations, along with those included in their more closely related clusters (XO, JB, A, XG, R, P, Q, XQ populations) are mostly wild materials, with only populations A (Turkey) and XO (India) being of uncertain biological status. Interestingly, they were assigned to cluster P1 (the only exception is population XQ), they carried the allele ‘A’ at the locus CAP and showed a high variability at the locus cpSSR with the presence of all the four alleles (Fig 6).

## Discussion

The main aim of the present study was to characterize, through both morphological evaluation and genetic diversity analyses, a large sample of worldwide distributed wild and cultivated *Poa pratensis* encompassing 23 different countries of Europe, Asia, America and Africa. The gained knowledge about their morphology, DNA content, level and structure of genetic diversity and genetic relationships are important both for interpretations of the evolutionary history of *P*. *pratensis*, while also providing useful data for breeders.

The analysis of the morphological data showed that the most relevant traits able to distinguish cultivated from wild forms of *P*. *pratensis* were spring growth habit and leaf colour (Table 4). This was confirmed both considering these traits independently, as well as considering them together, where both traits resulted correlated with the first two discriminant functions able to explain 52% of total variation. These traits are some of the most important traits that underwent selection during domestication, first, and breeding later [50]. In particular, rust tolerance, prostrate growth habit and dark green colour are among the most important traits selected by breeders for any new turf variety [50]. In fact, the dark green colour and rust tolerance are commonly associated with a healthy turf, while the prostrate growth habit with a better ground cover. Moreover, shorter basal internodes (short stature), higher tiller density, narrow leaf blades, high ground cover, all characteristics related to growth habit, were pursued since domestication [50,51]. Thus, our phenotypic analyses provide evidence that breeding selection for growth habit and leaf colour contributed to the cultivated phenotype. In contrast, traits such as inflorescence pigmentation and flag leaf length were apparently not influenced by anthropic selection pressures, as individuals with extreme values were found almost equally distributed between wild and cultivated forms (Table 4).

In recent years, flow cytometry has become the preferred technique for estimating nuclear DNA content because of its ease of utilization and accuracy [52,53]. The DNA content values found in this study were similar to those reported by other authors. Studying 38 accessions of a USDA core collection of Kentucky bluegrass, Wieners and collaborators [10] observed values ranging from 4.85 to 15.70 pg. In a different study on twenty-two commercial cultivars of this species, values ranging from 5.39 to 17.69 pg were reported [18]. The continue distribution of DNA content (S1 Fig) of the studied collection suggests the presence of several aneuploids in both wild and cultivated *P*. *pratensis* accessions and confirm the high genome variability of this species [10,18]. In fact, as suggested in previous studies DNA content can be efficiently used as a means to determining ploidy level in Kentucky bluegrass [18].

Genome size estimation in a polyploid species can be very important since molecular markers alone can fail in detecting the diversity caused by gene dosage effect, which may, in turn, affect the phenotypic diversity between accessions. Accordingly, we identified correlations between genome size and morphological traits related to both plant morphology, growth habit and rust tolerance in both wild and cultivated *P*. *pratensis* accessions. This supports previous studies which showed that variation of nuclear DNA content is associated with morphological characters in different plant species, for example negative correlations between nuclear DNA content and mean leaf length and width have been reported in *Dasypyrum villosum* (species of annual grass in the Poaceae family) [54]. The overall flow cytometry data produced in this study revealed the existence of both intra-and inter-population variation in *P*. *pratensis* DNA content. As suggested by Mowforth and Grime [55] intra-population variation in DNA content can arise through opposing forces: i) natural selection acting to reduce DNA amount, ii) the disposition of some genomes to accumulate “selfish” DNA, and iii) the existence of ecological advantages or disadvantages associated with large and small DNA amounts. The same authors postulated that in in *P*. *annua*, individuals with large genomes have an advantage under winter and early spring conditions, sufficient to guarantee their survival compared to faster-growing individuals characterized by small genomes. The negative correlation between genome size and spring-regrowth detected in this study may suggests a similar role for the DNA content in the distribution of *P*. *pratensi*s, with respect of climate and growing season length. On the other hand, the presence of individuals with contrasting genome size in cultivated accessions (Fig 3), subjected to intense breeding selection, as documented in this study may also suggest the natural disposition of *Poa* genome to accumulate DNA. This tendency is even more clearly evident in wild populations where as many as 10 out of 16 (62.5%) were found to be characterized by at least two ploidy levels. In fact, as early suggested by Bashaw and Funk, the great adaptiveness of this species could be closely related to its variable ploidy and by the presence of asexual reproduction that provides an escape from the typical reproductive problems associated with unbalanced genomes [4].

Chloroplast genes have been used in numerous phylogenetic studies and have proven to be useful in distinguishing different species belonging to the same genus. Patterson and collaborators used chloroplast markers to dissected the relationships among different species of the complex *Poa* genus [56]. Data produced in this study on both wild and cultivated *P*. *pratensis* accessions, showed low polymorphism level in the *trn*L-F region. This condition was suggested by Stoneberg Holt and collaborators [57] studying three *P*. *pratensis* varieties. However, our data (S1 File), originated from a world wild collection, allow to speculate a situation of widespread homogeneity in this species.

The low diversity here observed could be both related to the low nucleotide substitution rate in plastid DNA and to the mode of reproduction of the species. In fact, the existence of two different asexual reproduction mechanisms (i.e. apomixis and rhizomes production) could strongly reduce plastid diversity within accessions that can derive from one or very few maternal individuals clonally propagated. It is noteworthy that wild and cultivated accessions were well separated according to the allelic state of the SNP position identified in the chloroplast sequence suggesting a strong phylogenetic value for polymorphisms in this region.

Differently from the chloroplast analysis, the high level of polymorphism observed at the nuclear level confirms the suitability of SSR markers in genetic studies of Kentucky bluegrass. In fact, the number of alleles detected per SSR locus (from 1 to 18) was similar to that reported by Honig and collaborators (7–25) [26]. The high level of genome variability here reported, in terms of somatic DNA content and SSR allelic diversity, is not surprising considering the high variable ploidy level repeatedly documented for the species [10,18], the coexistence of apomictic and sexual reproduction in wild populations [7,9] and the pollen recognition system, which confers a strong ability to hybridize and retain alien genomes [58], all factors contributing to the extreme complexity of the genome of this species [59].

Only a modest relationship between genetic divergence and geographical origins was reported in *P*. *pratensis* by several authors [22,23]. A general lack of separation between cultivated and wild materials at molecular level was reported by Johnson and collaborators [23], who evaluated a USDA *P*. *pratensis* collection using RAPD markers and found a wide overlap of 17 commercial cultivars with clusters formed by wild accessions. Our results are consistent with these findings; indeed both the NJ and population structure analyses indicated that a clear distinction between wild and domesticated was not found (bootstrap values in NJ are almost all lower than 50% and, for STRUCTURE, wild and domesticated were attributed to both the two clusters, even if domesticated were mostly attributed to P2). The main reason of a similar genetic structure could rely on breeding activities used to develop new cultivars of Kentucky bluegrass starting from ecotypes. Finally, the widespread distribution of some ecotypes can generate confusion on the improvement status and on the geographical origin of some accessions that can be wrongly considered native of a given place.

However, the analyses of both chloroplast and nuclear data highlighted some differences between wild and cultivated materials. In particular, considering the chloroplast cpSSR a strong reduction of variability was detected in cultivated materials, where only two alleles were conserved out of the four detected in wild accessions. Concerning the chloroplast locus *trn*L-F most of the cultivated accessions showed the ‘C’ allele (82%), while, by contrast a predominance of the ‘A’ allele (71%) was found for the wild accessions. A higher variability was present at nuclear level for wild forms, where the two different clusters were equally represented (average q_P1_ = 0.51 and q_P2_ = 0.49), while the domesticated forms were mostly belonging to cluster P2 (average q_P2_ = 0.79), which showed also a significantly lower genetic diversity compared to the cluster P1. Thus, in spite of specific characteristics of the species that, as mentioned above, could make difficult a distinction between wild and domesticated forms, effects of domestication and breeding, were detected at molecular level by our analysis, with a certain loss of genetic variability in domesticated forms as expected due to founder effects and selection at the target loci.

The analyses at molecular level showed also interesting results related to the origin of the species. In this regard, we first focused our attention on the NJ tree. It showed the relationships between the different populations, with the main result being a clear and well supported subdivision, due to the 100% bootstrap value between the two wild populations from Asia (Mongolia XD and China, XC) and all the others. These two populations were included in cluster P1, the one with a lower F_K_ estimate compared to cluster P2 [34]. F_K_ parameter is similar to F_ST_ but it is specific for each population and is expected to be proportional to the divergence from a common ancestral population, thus suggesting a more ancient origin of P1 germplasm. Moreover, Mongolian and Chinese populations carried the ‘A’ allele at the chloroplast locus *trn*L-F, the most represented allele in wild forms and also among the individuals assigned to cluster P1. All these outcomes seem to indicate China and Mongolia as the most probable geographical areas of origin of the species.

Population XO (India) and JB (USA, NJ) were those most closely related to China and Mongolia in the NJ tree, even if their position was not statistically supported by bootstrap. Although both populations belong to cluster P1 and carry the ‘A’ allele, JB is a cultivated form and XO is of uncertain biological status, hence it is difficult to clearly understand their role and origin.

Within the limits of resolution of the present study, the molecular evidences here presented suggest Mongolia and China as the most probable center of origin of *P*. *pratensi*s. However, further studies, including a wider sample of *P*. *pratensis* accessions from Asia would be needed to obtain a more detailed picture on the topic.

## Supporting Information

S1 Fig Nuclear DNA content distribution. Histograms representation of Nuclear DNA content (pg/2C) distribution of successfully characterized *P*. *pratensis* accessions.
(TIF)

S2 Fig *trn*L-F chloroplast region sequences alignment. Portion of the *trn*L-F sequences alignment showing both 5bp indel and SNP position. In the alignment, *P*. *pratensis* individuals names are built by population name and individuals number (*i*.*e*. B29 is individual 29 from population B). For reference sequences Gene Bank accession number is reported.
(TIF)

S3 Fig Inference of the most probable K-value. a) Average *ln* likelihood value over 10 runs ± SD for increasing K-values, from 1 to 10. b) ΔK values over 10 runs for increasing K-values, from 2 to 9. c) Average symmetric similarity coefficient (H’) for K from 2 to 10.
(TIF)

S1 File SSR, cpSSR and LocusCAP dataset. (XLSX)

S1 Table Genetic diversity estimates for the 33 *P*. *pratensis* populations. (DOCX)
