# Supplementary figures and images for: Understanding Genetic Diversity and Population Structure of a Poa pratensis Worldwide Collection through Morphological, Nuclear and Chloroplast Diversity Analysis
Source: PLoS One. 2015 Apr 20;10(4):e0124709. doi: 10.1371/journal.pone.0124709 (PMC4404055; doi:10.1371/journal.pone.0124709)

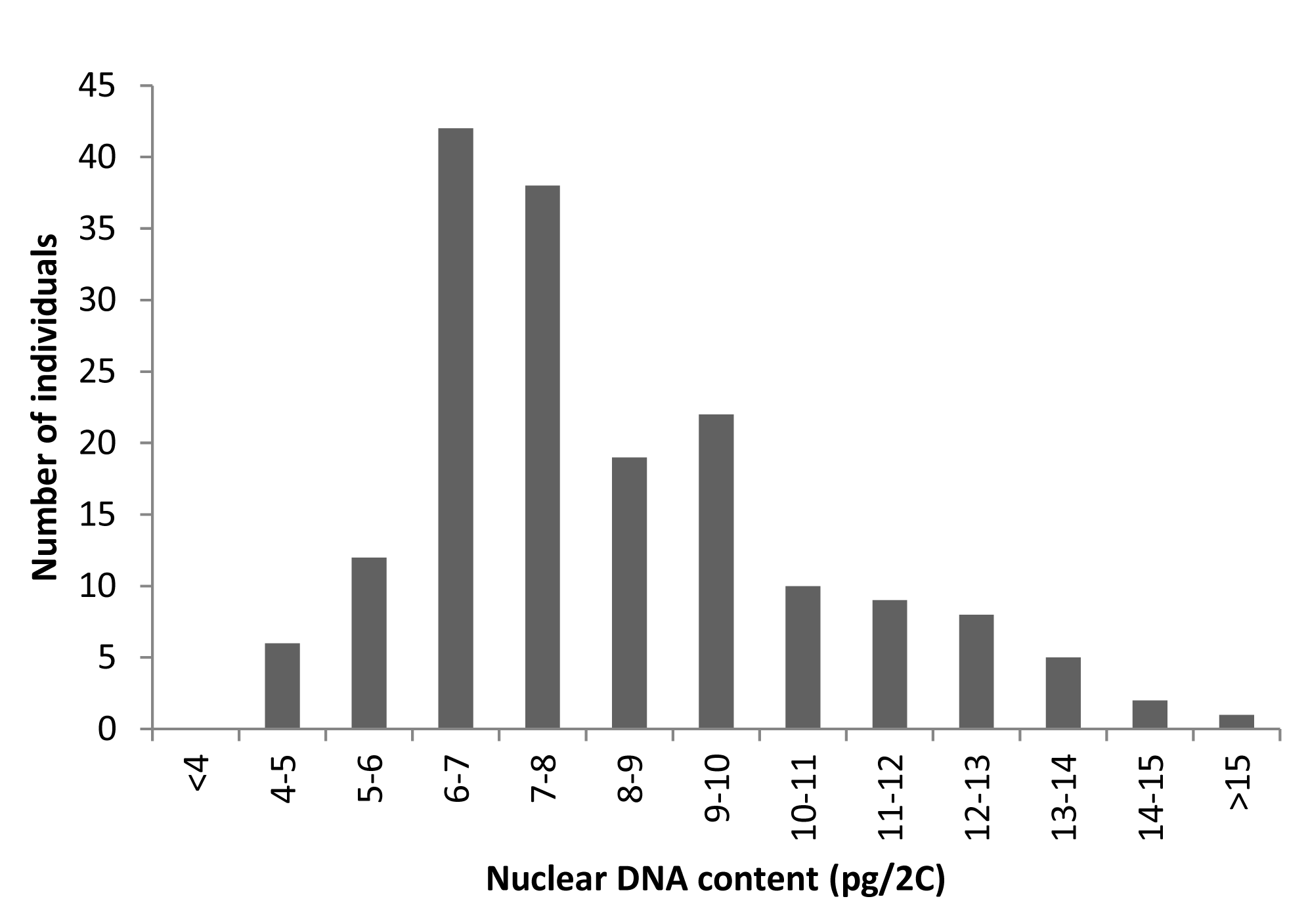

Supplement: S1 Fig — Histograms representation of Nuclear DNA content (pg/2C) distribution of successfully characterized P. pratensis accessions. (TIF) [file pone.0124709.s001.tif]

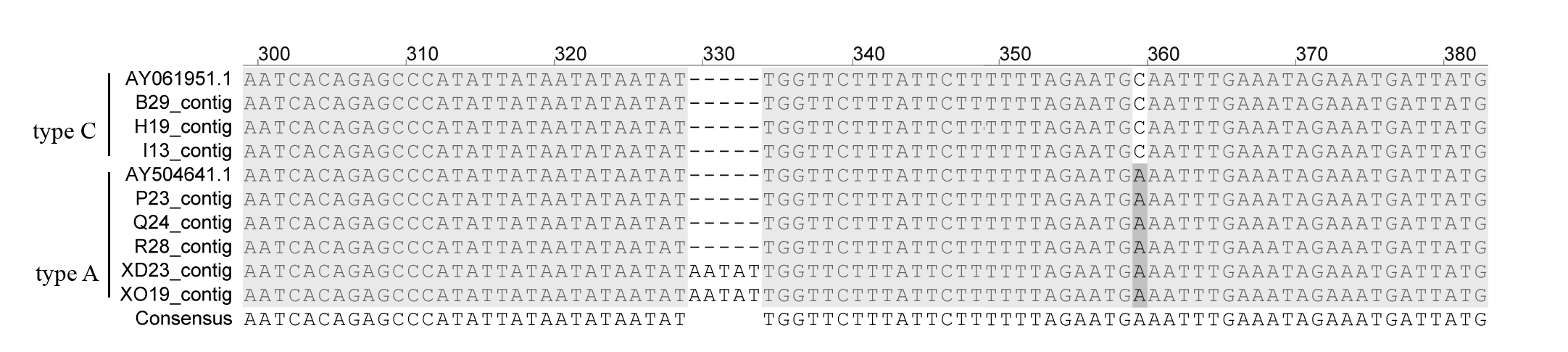

Supplement: S2 Fig — Portion of the trnL-F sequences alignment showing both 5bp indel and SNP position. In the alignment, P. pratensis individuals names are built by population name and individuals number (i.e. B29 is individual 29 from population B). For reference sequences Gene Bank accession number is reported. (TIF) [file pone.0124709.s002.tif]

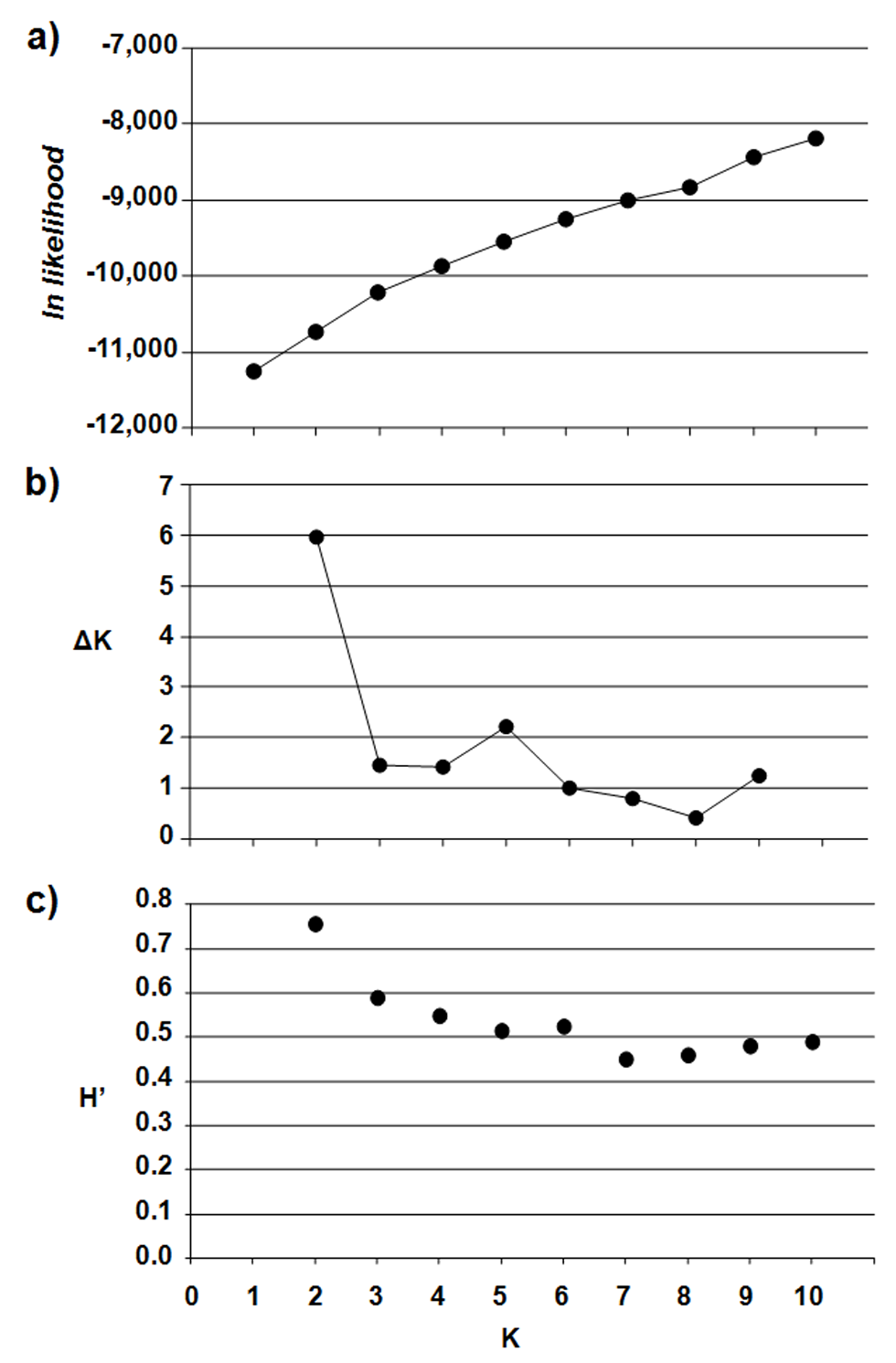

Supplement: S3 Fig — a) Average ln likelihood value over 10 runs ± SD for increasing K-values, from 1 to 10. b) ΔK values over 10 runs for increasing K-values, from 2 to 9. c) Average symmetric similarity coefficient (H’) for K from 2 to 10. (TIF) [file pone.0124709.s003.tif]
