# Supplementary material for: Understanding Genetic Diversity and Population Structure of a Poa pratensis Worldwide Collection through Morphological, Nuclear and Chloroplast Diversity Analysis
Source: PLoS One. 2015 Apr 20;10(4):e0124709. doi: 10.1371/journal.pone.0124709 (PMC4404055; doi:10.1371/journal.pone.0124709)
Supplement: S1 Table — (DOCX) [file pone.0124709.s005.docx]

S1 Table. Genetic diversity estimates for the 33 *P. pratensis* populations.

| # | Accession name | Geographic origin | Biological status | LocusCAP | Hj^†^ | SE (Hj)^§^ |
| --- | --- | --- | --- | --- | --- | --- |
| 1 | A | Turkey | Un | A/C, 0.75 | 0.24AB | 0.016 |
| 2 | B | Sweden | C | C | 0.24AB | 0.015 |
| 3 | E | Iran | W | A/C, 0.13 | 0.23AB | 0.014 |
| 4 | H | Netherland | C | C | 0.20AB | 0.015 |
| 5 | I | Poland | Un | C | 0.22AB | 0.014 |
| 6 | L | Germany | Un | C | 0.21AB | 0.015 |
| 7 | M | Hungary | L | C | 0.24AB | 0.016 |
| 8 | N | Denmark | C | A/C, 0.75 | 0.24AB | 0.016 |
| 9 | O | Belgium | C | C | 0.26A | 0.016 |
| 10 | P | Italy (PG) | W | A | 0.21AB | 0.015 |
| 11 | Q | Italy (IS) | W | A | 0.18B | 0.012 |
| 12 | R | Italy (VE) | W | A | 0.21AB | 0.014 |
| 13 | S | Italy (NO) | W | A/C, 0.25 | 0.22AB | 0.015 |
| 14 | T | Italy, (MC) | W | A/C, 0.38 | 0.24AB | 0.015 |
| 15 | V | USA (NC) | C | C | 0.20AB | 0.012 |
| 16 | JB | USA (NY) | W | A/C, 0.88 | 0.24AB | 0.016 |
| 17 | JD | USA (IL) | C | A/C, 0.13 | 0.23AB | 0.016 |
| 18 | JE | USA (AK) | C | A | 0.20AB | 0.013 |
| 19 | JF | USA (OR) | C | C | 0.18B | 0.012 |
| 20 | JG | Italy (RI) | W | C | 0.25AB | 0.017 |
| 21 | XA | Japan | C | A/C, 0.13 | 0.23AB | 0.015 |
| 22 | XC | China | W | A | 0.25AB | 0.018 |
| 23 | XD | Mongolia | W | A | 0.19B | 0.012 |
| 24 | XG | Russian Federation | W | A | 0.24AB | 0.016 |
| 25 | XI | Ukraine | W | A/C, 0.13 | 0.23AB | 0.017 |
| 26 | XL | Afghanistan | W | A | 0.23AB | 0.016 |
| 27 | XM | Czech Republic | Un | A | 0.20AB | 0.015 |
| 28 | XN | United Kingdom | C | C | 0.20AB | 0.014 |
| 29 | XO | India | Un | A | 0.23AB | 0.016 |
| 30 | XP | Morocco | W | A | 0.18B | 0.011 |
| 31 | XQ | South Africa | W | A | 0.18B | 0.011 |
| 32 | XR | Spain | W | A | 0.21AB | 0.014 |
| 33 | XU | Canada | W | A/C, 0.38 | 0.23AB | 0.015 |

† Hj, gene diversity estimate, analogous to the unbiased expected heterozygosity He (Nei 1978); Hj, values followed by different letters are significantly different at P<0.05.^§^ SE, standard error.
